# Birth rates and migration: an AI and One Health approach within the Italian National Health System

**DOI:** 10.3389/fpubh.2026.1790700

**Published:** 2026-03-26

**Authors:** Francesca Zappacosta, Balázs Majtényi, Alessio Laconi, Giovanni Di Nardo, Pasquale Parisi, Daniela Polese

**Affiliations:** 1 Department of Political Sciences, Sapienza University of Rome, Rome, Italy; 2 Department of Human Rights and Politics, Faculty of Social Sciences Eötvös Lorant University (ELTE), Budapest, Hungary; 3 Research Affiliate CNR-ISGI, Rome, Italy; 4 Department of Law, University of Florence, Florence, Italy; 5 Chair of Pediatrics, Department of NESMOS, Faculty of Medicine and Psychology, University of Rome Sapienza c/o Sant’Andrea University Hospital, Rome, Italy; 6 Department of Neuroscience, Mental Health and Sensory Organs NESMOS, Sapienza University of Rome, Rome, Italy; 7 UOD of Childhood Neuropsychiatry, Sant’Andrea University Hospital, Sapienza University of Rome, Rome, Italy

**Keywords:** demographic decline, Human Birth Theory, immigration, Italian national health system, one health approach, artificial intelligence (AI)

## Abstract

This study examines the future stability of Italy’s health and social systems in the context of demographic decline. The Italian healthcare system, grounded in a universalistic and public model, founded on the principle of equity, is undergoing a phase of instability. The COVID-19 pandemic represented a critical rupture, exposing systemic weaknesses, territorial disparities, infrastructural inadequacies, the rigidity of financing mechanisms and the lack of reform regarding citizenship rights for foreign residents. An inductive, interpretive approach was adopted, starting with data analysis from Italy’s population dynamics as of 1 January 2025, including births and deaths among citizens, growth of the foreign residents, and total social security contributions collected by the National Institute for Social Security. The analysis considered three major immigration reports, several academic articles from the legal, social, and medical fields, Constitutional Court Judgment No. 192/2024 on regional autonomy, and Mission 6 of the National Recovery and Resilience Plan within the framework of the Next Generation EU. The issuance of long-term visas and easier access to citizenship for foreign residents foster a more equitable society and can enhance the long-term sustainability of the Italian National Health System by offsetting demographic decline, lowering the median age, and stabilizing social security contributions. The success of such measures depends on integration policies and the efforts to combat racial discrimination. The findings highlight the need for citizenship and integration policy reforms and a governance framework consistent with the One Health approach. These measures would enhance the sustainability of Italy’s NHS and promote an equitable society.

## Introduction

The number of Italian citizens is expected to decline by approximately 8% by 2050, decreasing from 58,934,000 in 2024 to 54.4 million, driven by an aging population and a declining birth rate. By mid-century, more than 35% of Italians will be over the age of 65, while children under 14 will make up only 11.7% of the population. This demographic shift will place increasing pressure on the healthcare and social systems.

An editorial from early 2025 of *The Lancet Regional Health Europe* has expressed concerns, underscoring the pressing need for structural reforms to ensure the future stability of Italy’s health and social systems in the face of an aging population and demographic decline. This demographic shift will strain healthcare and social systems, also featured by a regional fragmentation. “Failure to act will deepen inequalities, delay treatments, and hinder progress—it has been highlighted—whereas prioritizing systemic reform offers Italy the opportunity to meet healthcare demands and deliver fair, efficient care” (1).

The present study considered interdisciplinary contributions exploring the normative and cultural dimensions of integration. In particular, the Human Birth Theory (2–6) has been adopted, as it examined the concept of human equality at birth, both from a scientific perspective and as a cultural enabler for the acceptance of diversity, thereby supporting integration policies that can foster population growth while ensuring the long-term sustainability of the healthcare system. This theory, being applied in Italy, supports the notion of equal access to social systems for all individuals, enabling the full realization of human potential to optimize societal functioning (4, 5, 7). A common brain activation and mind functioning at birth, independently from the ethnic and geographic origin, from culture and gender (8, 9), represents a robust base for the acknowledgement of a specific human nature, equal in all individuals at birth. Equality facilitates the integration of all individuals into society and their positive and productive interaction and it significantly influences both population growth and the utilization of services (10–12). To date, this is the first interdisciplinary study to apply this theoretical approach to equality in advocating for healthcare system reform, with the goal of making the system more equitable and sustainable.

Such an approach is considered essential for developing integration strategies that not only respond to demographic decline but also strengthen the sustainability and equity of the national healthcare system. The Italian National Health System (NHS), set up by Law 833 of 23 December 1978, is known to be universalistic and thus aimed at providing wide and equal access to healthcare services to every individual, regardless of residence or citizenship. Since the 1990s, the NHS has undergone reforms involving decentralization and privatization that, in name of containing public expenditure, have neglected its founding principles, resulting in a fragmented and unequal system that is not always able to provide universal healthcare services (13, 14). This trend continues to persist and exacerbates the above demographic problem, which could nevertheless be seen as an opportunity to revitalize the NHS in the face of the new multi-ethnic society that Italy is creating.

To provide a *pars construens*, it is essential to highlight ongoing trends of the Italian healthcare system, with particular attention to the impact of the migrant population, jurisprudential developments concerning it, and institutional and legal governance reforms that, if properly implemented or expanded, could mitigate the abovementioned issues.

In this study, a cross-sectional analysis was conducted to examine the future stability of Italy’s healthcare and social systems in the context of demographic challenges. The analysis was based on data from the National Institute of Statistics and national research centers that have the aim of informing government decision-making.

Complementing the quantitative analysis, an extensive investigation of both legislative texts and court rulings relevant to the sustainability of the national health system was undertaken, alongside a review of academic literature. This literature focused on the institutional and regulatory frameworks governing healthcare systems in relation to the implementation of the One Health approach, as well as migrant integration policies within EU Member States, with particular emphasis on the Italian context. Moreover, the investigation included research that interprets migration through a systemic and socio-economically positive lens, highlighting the potential of inclusive integration strategies to enhance social cohesion, economic vitality, and demographic renewal.

## Methods

An inductive, interpretive approach was employed, starting with data analysis to develop insights into the phenomenon of interest. Specifically, the study examined Italy’s population dynamics as of 1 January 2025, focusing on birth and death rates among Italian citizens, the increase in foreign residents, the issuance of new permits to non-EU citizens, and total social security contributions collected by the National Institute for Social Security. Additionally, three key reports from national research centers on immigration dynamics, relevant for policymakers, were analyzed.

Given the regional fragmentation of the healthcare system, the study also considered Constitutional Court Judgment No. 192 of 3 December 2024, which addresses differentiated regional autonomy. Furthermore, the research assessed Mission 6 of the National Recovery and Resilience Plan (NRRP) under Regulation (EU) 2021/241 of February 21, 2021, as well as the implementation status of the EU AI Act within the national legal framework.

In Italy, the principle of equity within the healthcare system is legally grounded in Article 32 of the Constitution, which protects health as a fundamental right of the individual and a collective interest, guaranteeing free medical care to those in need. This principle translates into the obligation to ensure all citizens—regardless of their social or economic status—equitable access to healthcare services and provisions, with due consideration for individual health needs.

The Italian National Healthcare System is universal in nature, meaning that healthcare services are extended to the entire population. As such, it seeks to implement the fundamental principles enshrined in the Italian Constitution. Healthcare services must therefore be provided without distinction or discrimination, ensuring equal access for all individuals with equivalent health needs.

Equity is a core principle of the system and refers to the necessity of allocating resources and services in a manner that guarantees fair access to care, taking into account individuals’ specific health conditions and varying socio-economic contexts. To this end, the Essential Levels of Care (Livelli Essenziali di Assistenza, LEA) have been established in accordance with Article 117 of the Constitution. The LEA define the healthcare services and provisions that the SSN is obliged to offer all citizens, either free of charge or subject to the payment of a nominal fee (ticket), in order to ensure equitable access.

In this context, the study considers the Constitutional Court’s ruling on the constitutionality of the provisions contained in Law No. 86 of 26 June 2024, concerning the implementation of differentiated regional autonomy. The ruling clarifies that while regions may request additional competences, including in the area of healthcare, such implementation must comply with the fundamental principles of the Constitution, including those underpinning the NHS.

Furthermore, the reforms encompassed within the projects funded by the National Recovery and Resilience Plan within the framework of the Next Generation EU (NGEU), specifically relating to the healthcare sector, were analyzed. These reforms aim to address the increasing structural instability of the healthcare system through the deployment of artificial intelligence and, notably, the integration of the One Health approach into the national system and governance framework.

## Results

As of 1 January 2025, Italian citizens have declined to 58.93 million (−0.6% annually), with only 370,000 births recorded in 2024. The foreign resident population has risen to 5.42 million (9.2% of the total), up +3.2% year-on-year, while the number of Italians residing abroad exceeded 6 million (15). This demographic shift continues to strain healthcare, pension, and social welfare systems, with a shrinking workforce supporting growing older population. According to the latest report from the Leone Moressa Foundation, the average age of immigrants in Italy is 35.7 years, compared to 46.9 years for Italians. Moreover, birth rates among immigrant communities are significantly higher, at 10.4 births per thousand inhabitants, compared to 6.3 for Italians (16).

In 2023, 3,059,431 foreign workers in Italy made a significant economic contribution, accounting for 8.8% of total national social security revenues (€16.4 billion). This also extends to tax revenues—immigrants declared 72.5 billion euros in income and paid 10.1 billion euros in Personal Income Tax (IRPEF), which directly supports national healthcare (17).

In 2023, the Reception and Integration System (SAI—*Sistema di Accoglienza e Integrazione*, established by Decree-Law no. 130 of 21 October 2020) served 54,512 beneficiaries, marking a 2.4% increase in admissions compared to the previous year. The majority of these individuals were included in ordinary reception projects (76.9%), while 21.2% were minors in dedicated programs, and 1.9% were individuals requiring mental health and specialized healthcare services—a category that grew by 25% compared to the previous year (18).

Additionally, the National Coordination of the New Italian Generations (CoNNGI) is composed of 40 associations founded by young people of migrant backgrounds and serves as a bridge between Italy and 40 other countries. By collaborating with institutions, universities, and NGOs, CoNNGI fosters professional development and innovation, ensuring that young migrants become an integral part of Italy’s future. The benefits of these initiatives are highlighted in the IDOS Report and ISTAT data.

Concerning the regional fragmentation of the healthcare system, in its ruling of 3 December 2024, the Constitutional Court annulled seven provisions of Law No. 86/2024 on differentiated autonomy—the new piece of law enabling regions to negotiate expanded competences in areas such as healthcare, education, and environmental protection—for violating constitutional principles. The Court stressed the need for uniformity in healthcare planning, infrastructure, and digital systems, reaffirming that equality and the right to health require: standardized essential services (LEP); centralized fiscal responsibility, and subsidiarity conditioned on demonstrable improvements in governance in order to ensure full compliance with the Italian Constitution (19).

## Discussion

The findings indicate that immigration can help rejuvenate Italy’s aging population and sustain the labor force. From an economic perspective, immigrants make a substantial contribution to public wealth as they significantly contribute to the GDP of the country while placing less of a burden on the welfare system (20, 21). Evidence shows that immigrant residents in Italy reduce per capita healthcare expenditure (22), and despite the heterogeneity of the regional health systems, they can alleviate fiscal burden caused by the aging of the population (23). By virtue of these data, and notwithstanding the complexities of the Italian legal system, there are several best practices in migrant integration that should be further expanded. One of the most relevant examples is the Reception and Integration System (Sistema di Accoglienza e Integrazione, SAI). The SAI system consists of a network of local authorities that, within the limits of available resources, access the National Fund for Asylum Policies and Services to implement integrated reception projects. At the territorial level, local authorities, with the valuable support of third-sector organizations, provide integrated reception services that, in addition to offering food and accommodation, also include complementary measures such as information, guidance, assistance, and support, through the development of individual pathways for socio-economic integration. It plays a fundamental role in providing support to immigrants, facilitating their access to employment, housing, education, and social services.

Additionally, the National Coordination of New Italian Generations CoNNGI plays a crucial role in integration. CoNNGI, established in 2017 by a group of associations representing the new generations, has the aim of giving voice to their concerns and bringing them to the attention of key actors in society. It brings together a network of associations rooted across the country, placing at the forefront the active role of young Italians with a migrant background (23). Key solutions to counterbalance Italy’s demographic crisis foresee the promotion of forward-looking immigration policies that on the one hand facilitate the issuing of long-term visas to migrants and on the other allow for a broader granting of Italian citizenship. Immigrant-friendly policies, including an inclusive approach to visa issuance, are more likely to meet the health needs of migrants and the related health determinants, such as health literacy, reduction of stigma and discrimination (24). The universality of a healthcare system, if considered through the lenses of the One Health approach, is related to the inclusiveness of migration policies, and these should lead toward a stable integration of the immigrants and possibly to the granting of the citizenship which should alleviate the inequalities in the access to the services. Moreover, granting Italian citizenship to young foreign residents supports the long-term sustainability of the healthcare system by promoting integration and equal access to the labor market and increasing social security contributions. By 2045, more than one-third of the Italian population will be over the age of 65. At the same time, a growing shortage of doctors, nurses, and social workers is expected, with increasing difficulties in replacing those retiring (25). However, the success of citizenship reform depends on the effectiveness of immigrant integration policies (26, 27). If current integration initiatives maintain a positive trajectory, they will facilitate this process. A comparative analysis between Italy and Germany on the granting of citizenship to migrants indicates that simplifying naturalization procedures can serve as an effective strategic measure to mitigate the effects of demographic decline. Although both countries have experienced similar demographic trends, Germany has pursued substantial reforms to its citizenship laws since 1999, with ongoing efforts to enhance accessibility. By contrast, Italy’s naturalization process remains notably inflexible. The findings highlight the relative success of the German model and underscore the complexities Italy faces in addressing low birth rates (28).

### Addressing fragmentation in Italy’s health data system

A major issue in Italy’s healthcare system is regional fragmentation, exacerbated by the country’s extensive regional autonomy. While regional autonomy offers numerous advantages, it should be applied in a way that does not compromise healthcare access for the most disadvantaged part of the population. The disparity in life expectancy between northern and southern regions, with Trentino-Alto Adige having the highest and Campania the lowest, illustrates the inequities in access to healthcare services.

The recent ruling of the Constitutional Court on the *Autonomia Differenziata* law has significantly reduced the legislative scope of regional autonomy regarding the healthcare system, re-affirming constitutional principles of equality and adequacy and potentially addressing both Italy’s fragmented regulatory framework and health data system.

This decision is crucial for ensuring greater interoperability between regions, facilitating the effectiveness of electronic health records (EHRs), and standardizing healthcare services nationwide. A key future best practice could be the full implementation of the National System for the Prevention of Health Risks from Environmental and Climatic Factors (Sistema Nazionale Prevenzione Salute dai rischi ambientali e climatici, SNPS), which has been regulated by ministerial decrees in 2022 and 2023. The establishment of the SNPS was envisaged as part of the institutional governance reforms for public health prevention set out in the National Recovery and Resilience Plan (NRRP), within the framework of the Next Generation EU (NGEU) under Regulation (EU) 2021/241 of 21 February 2021. It was financed through the corresponding Mission 6, with the strategy also aiming to manage health data flows via new digital infrastructures, in line with the One Health approach (29). While primarily focused on prevention, SNPS also plays a crucial role in the integration of digital health data. Indeed, to enhance the country’s capacity, effectiveness, resilience, and equity in responding to current and future health impacts linked to environmental and climate risks, a series of synergistic investments has been outlined. These include the comprehensive strengthening of structures and services at national, regional, and local levels, as well as improvements in infrastructure, human and technological resources, and applied research (30, 31). Specifically, Mission 6, with a total allocation of €15.63 billion, covers a broad spectrum of interventions, ranging from community and hospital care to research, training, and data interoperability (32).

### The role of AI in healthcare governance and digitalization

Furthermore, investments in digital infrastructure under the NRRP continue to strengthen Italy’s national health system through One Health Approach. Additionally, AI holds significant potential to transform the Italian healthcare system by enhancing the quality of care, improving efficiency, and strengthening disease prevention, in line with the Quadripartite One Health guidelines, which emphasize the fair and equitable distribution of benefits arising from the use of AI and related digital technologies. Italy’s National Recovery and Resilience Plan identifies One Health as a central strategy for reducing health inequalities and ensuring the effective integration of AI into existing healthcare information systems.

Artificial intelligence (AI) can enhance healthcare by optimizing resource allocation, improving diagnostics, and reducing administrative burdens, which often hinder access for vulnerable groups such as the older population, migrants, and persons with disabilities. Tools such as telemedicine and advanced diagnostics are key to promoting equitable and timely care. In the context of migration policy reform, the implementation of the EU AI Act must align with national strategies and uphold human rights, particularly in regulating AI-driven immigration control systems as critical testing grounds for global AI governance (33).

Finally, adopting a “One Health Approach” within a “*Planetary Health*” vision (34) is essential for achieving effective multilevel healthcare governance and addressing the limitations exposed by the COVID-19 pandemic (35). This includes implementing structured prevention policies and enhancing the resilience of environmental governance, to strengthen the national health system from a sustainability perspective and underlining the connection between healthcare and environmental protection (36).

### The Human Birth Theory: a neurobiological cornerstone for equality and equity in public health policies

This study has identified the Human Birth Theory (4–6) as a framework capable of laying the foundations for equality and equity in public health for the general population in both developing and developed countries. Discrimination, in fact, can represent a serious obstacle to the reorganization of healthcare systems and public health, for both legislators and citizens.

To achieve these goals, a cultural shift is necessary, which is essential for integration policies and for overcoming the fragmentation of the national healthcare system. This is particularly important considering that the inclusion system—which recognizes migration as a long-term resource for Italy’s economic, social, and technological future—together with standardized healthcare governance, represents a crucial element in this process.

The Human Birth Theory, developed by the psychiatrist Massimo Fagioli (4), allows for the identification of a single species, defined by shared neurobiological characteristics. Discrimination is generally based on differences in skin color and physical appearance, which can vary from person to person. Essentially, it is based on the phenotype, namely what appears (from the Greek word *Fainomai* that means *to appear*) and not what essentially is. Despite these apparent differences in individual characteristics, the features of the human brain and its functioning at birth allow us to overcome phenotypic differences.

According to the proponents of the Human Birth Theory, brain functioning at birth is initiated by a universal mechanism: the separation from the mother’s body and the transition to a new environment. Specifically, the sudden exposure to light—previously absent in the intrauterine setting—stimulates the retina, triggering its neonatal activity and contributing to neurodevelopmental processes unique to humans.

Recent scientific studies have identified a non-visual photic pathway that is active from birth (37) and capable of transducing light into signals affecting the entire brain. This occurs via numerous retinal afferents projecting to both cortical and subcortical structures, likely resulting in widespread brain activation (9). Furthermore, a very recent study (38) has documented developmental changes over the birth transition, consisting in a dramatic surge of brain functional connectivity in newborns compared to the fetus.

According to proponents of the Human Birth Theory, this early light-induced brain activation, which occurs before the first breath (8, 9), coincides with the emergence of the mind. The neonatal mind exhibits species-specific characteristics, such as reactivity, vitality, annulment pulsion, capability to imagine and other (4). These dynamics characterize the human brain universally, regardless of ethnic, geographical or cultural background, thereby providing a scientific foundation for the principle of human equality. This has far-reaching implications for the recognition and protection of human rights, including equitable access to education, healthcare, and the promotion of physical, mental, and social well-being.

### Moving toward a sustainable and integrated future

To address Italy’s demographic and healthcare challenges, it is imperative to:

1. Expand access to citizenship and integration programs to sustain workforce numbers and counteract aging trends and to create a more equal society (39), adopting a scientific–medical theoretical approach for equality aimed at addressing and mitigating discrimination.
2. Strengthen the integration policies.
3. Implement structural reforms to unify Italy’s healthcare system, reducing regional disparities and improving data interoperability.
4. Promote AI-driven solutions, ensuring ethical and equitable implementation in compliance with European regulations.
5. Implement the One Health approach by effectively translating it into national law and governance to enhance its effectiveness.

## Conclusion

The findings offer a constructive perspective, highlighting several key areas for policy and legal reform and development.

The need for migrant integration reforms, including better access to citizenship, to promote social inclusion and equal opportunities.

The strengthening of institutional and legal frameworks for public health prevention, particularly enhancing the National System for the Prevention of Health Risks from Environmental and Climatic Factors.

The progressive alignment of artificial intelligence systems with the EU AI Act and the integration of public health policies with the ‘One Health’ approach, recognizing the interconnection between human health and environmental protection.

In this way, Italy’s healthcare system can remain sustainable despite demographic challenges.

## Data Availability

Publicly available datasets were analyzed in this study. This data can be found at: https://demo.istat.it/; https://esploradati.istat.it/databrowser/#/it/dw/dashboards.
